# A case report of idiopathic hyperaldosteronism characterized by bilateral adrenal adenoma

**DOI:** 10.1097/MD.0000000000017418

**Published:** 2019-10-25

**Authors:** Wei Wang, Feng Wei, RanHao Li, JiaHui Tian

**Affiliations:** Department of Endocrinology, First Affiliated Hospital of Baotou Medical Collage, Inner Mongolia University of Science and Technology, Baotou 014010, Inner Mongolia, China.

**Keywords:** bilater adrenal vein blood sampling, bilateral adrenal adenoma, idiopathic hyperaldosteronism

## Abstract

**Rationale:**

Primary aldosteronism (PA) comprises 2 main subtypes: unilateral aldosterone-producing adenoma (APA) and idiopathic hyperaldosteronism or named as bilateral adrenal hyperplasia (BAH). An accurate discrimination between APA and BAH is crucial because the former is treated with adrenalectomy (ADX) and the latter is primarily by aldosterone antagonists. We report a case of idiopathic hyperaldosteronism characterized by BAH.

**Patient concerns:**

A 46-year-old woman had experienced a paroxysmal elevation of blood pressure for the past 2 months, along with an intermittent headache and mild occipital swelling and pain.

**Diagnoses:**

We performed clinical, laboratory, and imaging tests, as well as bilateral adrenal vein sampling (AVS) on this patient. Specifically, computed tomography scan and magnetic resonance imaging were used to characterize the properties of bilateral adrenal adenoma. Additionally, bilateral AVS was performed to distinguish unilateral from bilateral adrenal abnormality in this patient.

**Interventions:**

After oral administration of aldosterone antagonists, her blood pressure and potassium levels returned to normal ranges and her condition improved.

**Outcomes:**

Following differential diagnosis, screening, functional tests, a variety of imaging studies, and bilateral adrenal vein sampling (AVS) typing, she was finally diagnosed with idiopathic hyperaldosteronism.

**Lessons:**

For PA patients with lack of typical hypertension and hypokalemia performance, early identification and accurate diagnosis are of great significance for improving the prognosis of BAH. AVS plays an important role in the classification of PA subtype, especially for the cases with bilateral lesions. In regard to patients with rare bilateral adrenocortical adenoma-type aldosteronism, AVS plays a key role in choosing the appropriate treatment regimen.

## 1 Introduction

Primary aldosteronism (PA) comprises 2 main subtypes: unilateral aldosteronism caused by aldosterone-producing adenoma (APA); and bilateral adrenal hyperplasia or known as idiopathic hyperaldosteronism (1). In both types, PA lesion may be located on one side or bilaterally. Adenomatous PA is mostly unilateral, multiple unilateral, or more rarely bilateral. Computed tomography (CT) and adrenal vein sampling (AVS) are recommended as the guidelines for the diagnostic work-up of patients with PA.^[1,2]^ Measurement of plasma aldosterone concentration and direct renin concentration (DRC) to calculate the aldosterone-to-renin ratio (ARR) is the most reliable method of screening for PA.^[3]^ An accurate discrimination between APA and bilateral adrenal hyperplasia (BAH) is crucial because the former is treated with adrenalectomy (ADX) and the latter is primarily by aldosterone antagonists.^[4]^ We report a case of idiopathic hyperaldosteronism characterized by BAH. In addition, this case did not present with a predominantly secretory side.

## 2 Case presentation

A 46-year-old Chinese woman was referred to the endocrine unit for further evaluation and management of hypertension (HTN). She initially experienced increased blood pressure (peak at 140/90 mm Hg), along with an intermittent headache and mild occipital swelling and pain for the past 2 months. After resting for approximately 10 minutes, her symptoms were relieved. The monitored blood pressure was approximately 125/85 mm Hg without headache. She did not receive diuretics or long-term medications. None of her immediate family members had HTN, stroke, or sudden death at a young age. Her father was diagnosed with HTN at 65 years of age.

No physical signs were found in the physical examination. Blood electrolyte analysis revealed a potassium concentration of 3.37 mmol/L and a synchronous 24-hour urine potassium concentration of 29 mmol/day, suggesting the possibility of potassium loss in the kidneys. The 24-hour urinary free cortisol level was 25.86 mmol/day, ranging from 4.2 to 35.5 mmol/day. Besides, the serum cortisol levels were 1.24 μg/day (2.5–15.8) at 0:00 and 8.81 μg/dL (4.26–24.85) at 8:00, while adrenocorticotropic hormone (ACTH) levels were 8.05 ng/L (4.6–36.3) at 0:00 and 8.51 ng/L (7.2–63.3) at 8:00. The cortisol-related data were all within the normal ranges, as similar to the blood and urine levels of catecholamine hormones (Table 1).

**Table 1:**
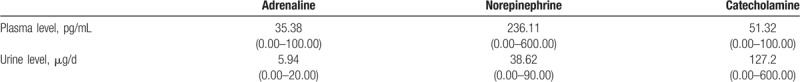
The levels of catecholamine hormones in the plasma and blood.

The levels of aldosterone and DRC were determined by an automated chemiluminescence immunoassay. According to the guideline,^[2]^ the values of aldosterone (ng/dL), DRC (ng/L), and ARR were calculated as 3.8, 5.7, and 7.7, respectively. These results were considered to be negative for PA, due to a low serum potassium level. After treating the low potassium, the screening findings appeared to be positive (Table 2). Our findings suggest that potassium levels should be within the normal range in order to adequately interpret the ARR. Thus, it is necessary to determine whether potassium levels are normal and to correct hypokalemia before performing the confirmatory tests.

**Table 2:**
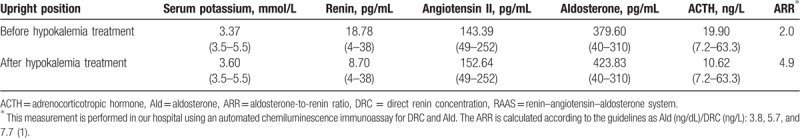
Changes in RAAS levels after 4 h of standing in the posture tests (2 tests).

Two confirmatory tests were performed, including captopril challenge test and saline infusion test. For captopril challenge test, the patient was orally administered with 50 mg captopril after 2 hours of sitting at 0700 hours. Blood samples were collected for the measurement of plasma aldosterone, DRC, and cortisol levels at 0, 1, and 2 hours after captopril administration, where the patient remained seated during this period. The results demonstrated that plasma aldosterone levels were suppressed by captopril (>30%), as shown in Table 3. For saline infusion test, the levels of aldosterone, DRC, and potassium levels were measured at 0800 hours (after 10 hours of bed rest). An infusion of 2000 mL of 0.9% normal saline solution was administered to the patient over a 4-hour period without standing. After 4 hours of infusion, the serum levels of aldosterone and DRC were measured. The results showed that the levels of aldosterone were >10 ng/day, exceeding the diagnostic cut-off value (Table 4). Based on the results of these tests, a diagnosis of PA was established.

**Table 3:**
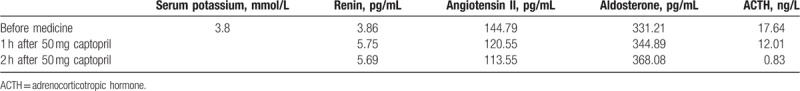
(Confirmatory test 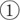): the results of captopril challenge test.

**Table 4:**
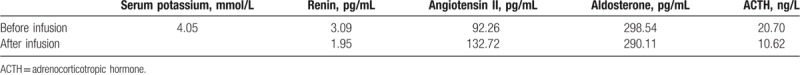
(Confirmatory test 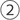): the results of saline infusion test.

For positional test, the serum levels of aldosterone, renin, and potassium were measured at 0800 hours (after 10 hours bed rest) and repeated after 4 hours of standing. As shown in Table 5, the levels of aldosterone were increased during upright positioning by 56.6% after 4 hours of standing, supporting a diagnosis of idiopathic hyperaldosteronism.

**Table 5:**
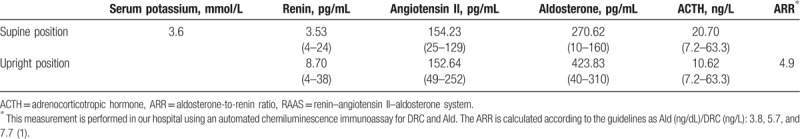
Positional test: RAAS test results in the supine and upright positions.

**Figure 1:**
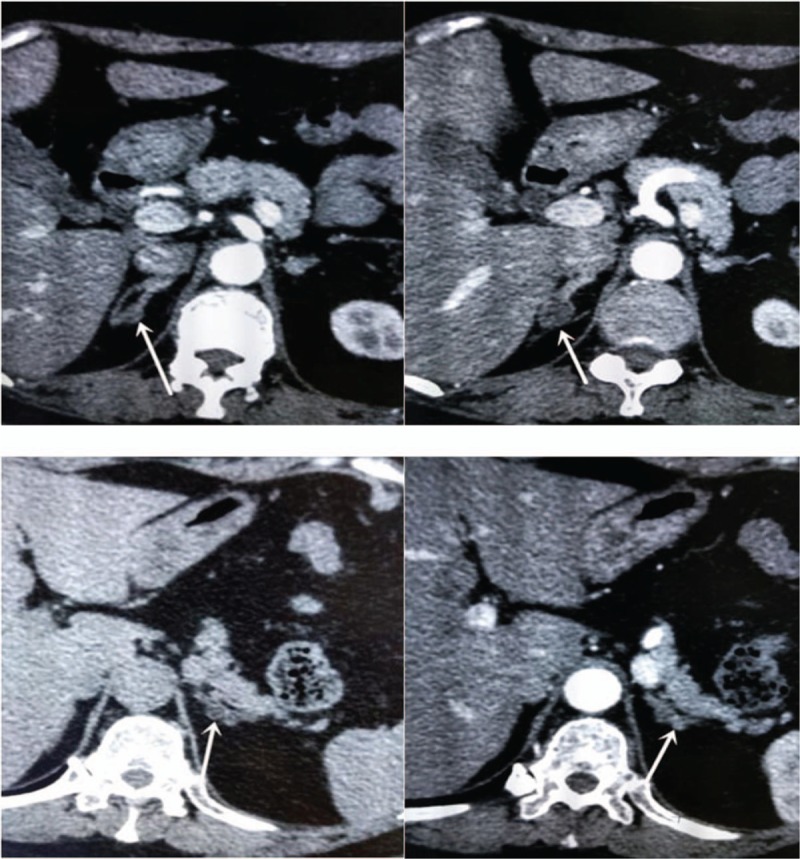
Bilateral adrenal enhancement computed tomography (CT) scan. The arrows indicate the bilateral adenomas.

Ultrasound examination of the adrenal gland revealed a low-echo area of approximately 1.8 × 1.5 cm in the right adrenal. Moreover, CT imaging of the adrenal gland revealed a bilateral adrenal gland in the multiple round shaped density shadow with clear borders. The largest one was visible on the right side of the medial limb, with a diameter of approximately 1.1 cm. Enhanced CT imaging of the adrenal gland indicted a smooth edge low-density shadow of approximately 1.2 × 1.1 cm in the right adrenal gland (Fig. 1). Further enhancement of CT imaging revealed a smooth edge low-density shadow of approximately 0.4 × 0.5 cm in the left adrenal gland (Fig. 1).

**Figure 2:**
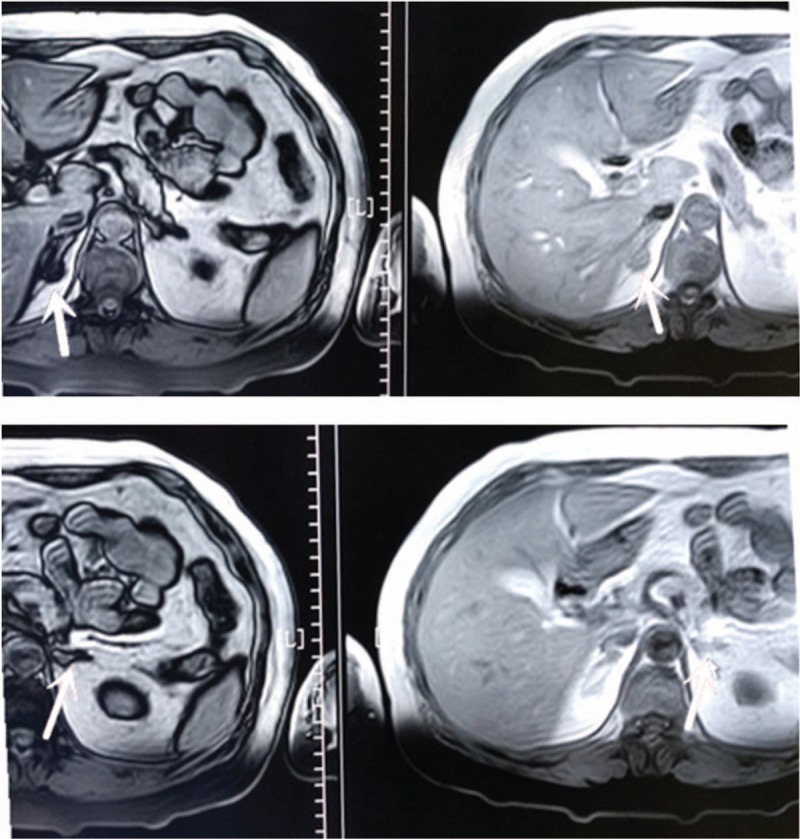
Bilateral adrenal magnetic resonance imaging (MRI) scan data showing the location of the bilateral adenomas.

Based on the results of opposed-phase chemical shift magnetic resonance imaging (MRI), the adenomas displayed bilateral adrenal thickening with multiple nodules, in which the right nodule was approximately 12.3 × 15.5 mm and the left nodule was approximately 12.8 × 10.6 mm (Fig. 2). Although early studies have reported that imaging techniques may be useful to locate APAs, the findings of CT and MRI are often discordant with those of AVS for PA subtype diagnosis.^[5]^

Bilateral AVS was carried out on this patient. Informed consent was obtained from the patient prior to sampling. For hormonal examination, bilateral blood specimens were collected from 6 different sites, including left adrenal vein, right adrenal vein, left renal vein, right renal vein, postcava at thoracic 12 level, and postcava at lumbar 2 level. After preoperative preparation, a necessary procedure was performed and the samples were immediately sent to the laboratory. The mean concentrations of aldosterone and cortisol were calculated to determine cortisol-corrected aldosterone ratio (PAC/PCC). As shown in Table 6, the hormonal levels of ipsilateral adrenal vein, renal vein, and inferior vena cava confirmed the successful intubation.^[6]^ Furthermore, the lateralization index for right/left is 1.1 (<2) indicated that there was no dominant side of secretion.^[6]^ Ultimately, the patient was diagnosed as idiopathic hyperaldosteronism.

**Table 6:**
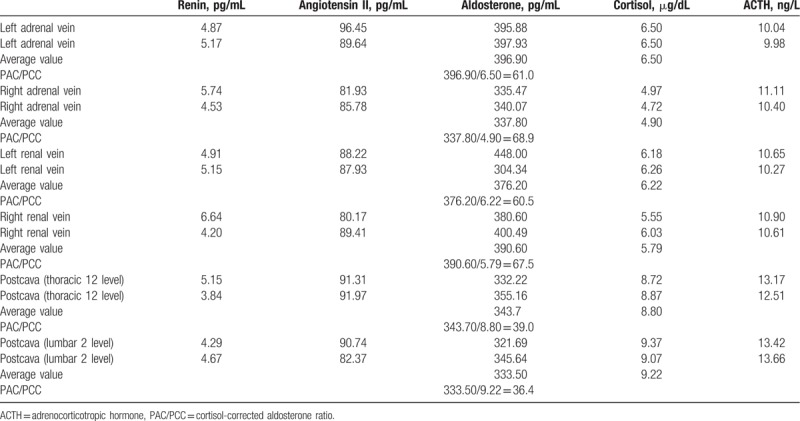
Results of bilateral adrenal vein blood sampling.

The treatment included 10 mg of spironolactone twice daily. After 1 week, her blood pressure was approximately 125/80 mm Hg, as monitored 4 times per day. Her serum potassium level was 3.59 mmol/L prior to discharge. One, 3, and 6 months after discharge, the patient's blood pressure was 120/80 mm Hg and blood potassium levels ranged from 3.62 to 4.17 mmol/L.

Ethical approval was not needed according to the rules and regulations of the hospital, as this case report did not involve any intervention.

Informed written consent was obtained from the patient for publication of this case report and accompanying images.

## 3 Discussion

PA was first defined and reported by Jerome W Conn in 1955. It is characterized by adrenal cortical secretion of aldosterone, leading to the inhibition of renin–angiotensin II–aldosterone system activity that is unrelated to sodium load. PA is the most common cause of secondary HTN, which responsible for 5% to 15% cases of increased blood pressure among hypertensive populations.^[7]^ In addition to its hypertensive effects, increases the risk of cardiovascular and cerebrovascular complications and metabolic syndrome, and exhibits proinflammatory actions on different organ systems.^[8,9]^ The estimated prevalence of secondary HTN is approximately 4% among primary care hypertensive patients and approximately 10% among referred patients.^[10]^ PA is particularly common in patients with resistant HTN, with a prevalence of 14% to 21%.^[11,12]^ In this case, the results of all examinations indicated a bilateral adrenal tumor, suggesting that the initial clinical assessment should consider the correlation between blood pressure and PA risk.

The underlying cause of PA in the majority of patients is either APA or BAH,^[9]^ together accounting for at least 95% of all cases. In general, APA and BAH account for approximately 40% and 60% of cases, respectively.^[13]^ PA caused by adrenal cortical adenoma is also known as adenomatosis, with subsequent high blood pressure occurring in approximately 0.5% to 2% of clinical cases. In the 2 major types of PA, the lesion(s) can be located on either one side or both sides. The majority of adenomatous proformaldehydes are single unilateral lesions, followed by multiple unilateral or bilateral lesions, and rare bilateral adenomas. Less than 2% of adenomatous aldehydes are reportedly present in bilateral adenomatous PA. BAH can occur bilaterally and is difficult to be distinguished from adenomas by imaging techniques. In this case, both refractory HTN and persistent hypokalemia were not presented. However, combined with its clinical features and imaging findings, BAH was included as a risk factor for the initial screening and early diagnosis of PA. This can be done under the guidance of general diagnosis and treatment. The special case of PA is further investigated for the etiology of proforma. Accurate identification of the subtype is essential for treatment decisions in patients with PA.^[1]^

APAs are mostly treated by ADX, while mineralocorticoid receptor antagonist is used to treat BAH.^[1,14]^ The correct classification and diagnosis was the major source of confusion and challenge in the present case. According to the American Endocrinology Society's 2016 guidelines for the diagnosis and treatment of PA,^[2]^ adrenal CT scan and AVS are recommended for differentiating between APA and BAH.^[15]^ CT examination is a noninvasive, painless, and relatively safe procedure, with a sensitivity 78% and specificity of 75%. However, the confidence level of CT is not high for the differential diagnosis of adenoma and hyperplasia. In this situation, bilateral adrenal nodules can be interpreted as bilateral hyperplasia on the basis of CT findings, but the patient may indeed have APA and adrenal incidentaloma on the contralateral side.^[15]^ A systematic literature review shows that both CT and MRI findings indicate the incorrect APA or BAH subtype in nearly 38% of PA patients, which may lead to an inappropriate treatment.^[16]^

In addition to those established methods, current guidelines have recommended AVS as the diagnostic workup of PA patients.^[1]^ The diagnostic accuracy of AVS is higher than that of CT, with 95% sensitivity and 100% specificity. At present, AVS is the only method recommended by the Endocrine Society guidelines to distinguish both subtypes and to justify whether the patients are eligible for ADX.^[2]^ In this case, bilateral adenomas were considered in multiple imaging examinations. Based on our previous experience, laparoscopic bilateral adrenal partial resection is an effective method for the treatment of bilateral functional adenomatosis. However, it remains unclear if this patient is clinically suitable to receive ADX.

AVS is considered as the gold standard method for subtype classification and determining whether elevated hormone secretion occurs on one particular site. One potential limitation of AVS is that bilateral APAs are not able to be distinguished from idiopathic hyperaldosteronism.^[17]^ Nevertheless, we believe that this limitation may only exist in 2 situations: when AVS indicates a predominantly secretory side and when adenomas are not obvious in the CT imaging, which may be necessary in both cases. In addition, segmental AVS was performed to clarify the segment of aldosterone produced inside the unilateral adrenal gland. It was confirmed that the detected bilateral APA was truly idiopathic aldosteronism. Through AVS, the lacking of predominantly secretory side within each adrenal gland should be diagnosed as hyperplasia, but not APA.

The theoretical basis for AVS is that the plasma levels of aldosterone in each adrenal vein are normalized to the concentrations of cortisol. Normal bilateral adrenal glands can produce the same amount of aldosterone and cortisol. The levels of cortisol are relatively constant in patients with PA, but aldosterone is secreted on the high side to suppress the opposite side.^[18]^ Notably, the concentration of aldosterone in adrenal vein is often higher than normal during PA lesion, and is easily diluted by other blood in adjacent blood vessels. Therefore, it is not possible to directly compare aldosterone levels, whereas cortisol production is uniform throughout the adrenal cortex, including the tumor-bearing region. As a result, PAC/PCC (aldosterone divided by cortisol level in its respective veins, namely the A/C ratio or PAC/PCC ratio) is calculated to assess the aldosterone lateralization and contralateral suppression and to correct the dilution effects. Additionally, to determine whether there is an obvious predominant secretory site that represents the same site of the lesion,^[15,17,19]^ AVS is performed by interventional intubation.

Due to the pulsed secretion of aldosterone, the stress during AVS procedure should be lowered to minimal level, the stress caused by the sequential method of blood collection may result in a gradient of hormone concentrations. This may influence the values of lateralization index, calculated by high side corrected aldosterone divided by low side corrected aldosterone.^[20]^ Therefore, simultaneous blood collection from the bilateral adrenal veins should be performed. In this study, the patient was asked to follow a strict preoperative procedure, and the bilateral synchronous intubation was simultaneously performed by 2 interventional physicians. Immediately upon collection, the blood samples were sent to the laboratory for testing in order to minimize the effects of stress and potential testing errors.

In overall, this patient is lacking of typical high blood pressure, clinical manifestations of hypokalemia, and characteristic imaging findings suggestive of BAH. However, patients with atypical symptoms often present a challenge in diagnosis classification and treatment, as these cases are required to adhere to strict guidelines. Therefore, an accurate diagnostic method is needed to avoid the risk of inappropriate surgical treatment and subsequent burden on patients, such as lifelong replacement therapy due to long-term adverse effects resulting from ADX. Although CT and positional test are not always reliable in subtyping these patients, they can serve as an important addition to the complicated diagnostic process.^[21]^ AVS should not be recommended for all PA patients but only for some rare cases of bilateral adrenal adenomatosis, especially the results of imageological examination and functional tests are in doubt. A previous radiological study also suggests that AVS is helpful when CT scan findings are equivocal or show bilateral disease.^[22]^ In short, AVS plays key roles in early diagnosis, selection of treatment, and disease management, especially for patients with rare bilateral adrenocortical adenoma-type aldosteronism. This technique can be used as a gold standard for imaging diagnosis of the suspected differential diagnosis.

## Acknowledgments

The authors thank the patient for allowing them to publish the case report and to use the images taken during her hospital admission.

## Author contributions

**Conceptualization:** Feng Wei.

**Data curation:** JiaHui Tian.

**Investigation:** JiaHui Tian.

**Resources:** RanHao Li.

**Supervision:** Wei Wang, RanHao Li.

**Writing – original draft:** Wei Wang, Feng Wei, RanHao Li.

**Writing – review & editing:** Wei Wang, Feng Wei.
